# Diplomatic advantages and threats in global health program selection, design, delivery and implementation: development and application of the Kevany Riposte

**DOI:** 10.1186/s12992-015-0108-x

**Published:** 2015-05-27

**Authors:** Sebastian Kevany

**Affiliations:** University of California, San Francisco, 550 16th Street, 3rd Floor, San Francisco, California 94158 USA

**Keywords:** Global health diplomacy, foreign policy, PEPFAR, HIV/AIDS, the Global Fund to Fight AIDS, Tuberculosis and Malaria, ‘smart power’

## Abstract

**Background:**

Global health programs, as supported by organizations such as the Global Fund to Fight AIDS, Tuberculosis and Malaria and the President’s Emergency Plan for AIDS Relief (PEPFAR), stand to make significant contributions to international medical outcomes. Traditional systems of monitoring and evaluation, however, fail to capture downstream, indirect, or collateral advantages (and threats) of intervention selection, design, and implementation from broader donor perspectives, including those of the diplomatic and foreign policy communities, which these programs also generate. This paper describes the development a new *métier* under which assessment systems designed to consider the diplomatic value of global health initiatives are described and applied based on previously-identified “Top Ten” criteria.

**Methods:**

The “Kevany Riposte” and the “K-Score” were conceptualized based on a retrospective and collective assessment of the author’s participation in the design, implementation and delivery of a range of global health interventions related to the HIV/AIDS epidemic. Responses and associated scores reframe intervention worth or value in terms of global health diplomacy criteria such as “adaptability”, “interdependence”, “training,” and “neutrality”. Response options ranged from “highly advantageous” to “significant potential threat”.

**Results:**

Global health initiatives under review were found to generate significant advantages from the diplomatic perspective. These included (1) intervention visibility and associations with donor altruism and prestige, (2) development of international non-health collaborations and partnerships, (3) adaptability and responsiveness of service delivery to local needs, and (4) advancement of broader strategic goals of the international community. Corresponding threats included (1) an absence of formal training of project staff on broader political and international relations roles and responsibilities, (2) challenges to recipient cultural and religious practices, (3) intervention-related environmental concerns, and (4) a lack of *prima facie* consideration of intervention diplomatic and foreign policy consequences.

**Conclusions:**

Global health interventions stand to generate significant diplomatic advantages for donor and recipient countries and organizations when appropriately selected, designed, targeted, and delivered. Conversely, in the absence of the application of standards such as those developed under the Kevany Riposte, threats to diplomacy and international relations may occur. With the application of related systems to other global health programmes and settings, comparative results on the relative worth of alternate approaches from the diplomatic perspective may be generated to better inform political, strategic, and global health policy and programmatic decisions.

**Electronic supplementary material:**

The online version of this article (doi:10.1186/s12992-015-0108-x) contains supplementary material, which is available to authorized users.

## Theoretical & philosophical basis

### Global health, diplomacy, and foreign policy

Foreign policy trends in both the European Union and the United States are increasingly aligned with “smart power” [1] approaches, in recognition of the “myriad newer uses” of global health and development initiatives [2]. The European Union advocates strategic use of foreign assistance, combined with diplomacy and arbitration, to pursue foreign policy goals under the Common Foreign & Security Policy [3,4] via a specific focus on global health [5]. Similarly, in the United States, increasing levels of alignment between the State Department, the United States Agency for International Development, the Department of Defense and the Department of Health and Human Services is indicative of their increasingly interchangeable roles [6]. In the United Kingdom, foreign assistance now forms an integral component of foreign affairs [7] under the stated goal of “policy coherence” [8]. Conversely but convergently, both the United Nations and international military forces have displayed an increased propensity to combine conflict resolution and humanitarian activities [9], whilst the World Bank has recommended that international development focus on security issues beyond primary programmatic goals [10]. These trends are of increasing importance to the design, delivery and evaluation of global health intervention programs under initiatives such as the United States’ President’s Emergency Plan for AIDS Relief (PEPFAR) [11] particularly in the context of the recent creation of the Office of Global Health Diplomacy [12] and related programs supported by the Global Fund to Fight AIDS, Tuberculosis and Malaria (“The Global Fund”) [13].

### Aligning & sensitizing global health programs to international affairs

Unlike the medically-dominated ethos of global health, diplomatic and foreign policy perspectives are both multilevel and multicausal, involving the synthesis of information from a variety of social science knowledge systems [14]. Failure to consider foreign policy and international relations principles and objectives when designing, selecting and implementing global health programs such as PEPFAR and the Global Fund’s related HIV/AIDS initiatives may, therefore, create a “tense and confusing duality” [15]. For example, while global health programs that challenge cultural, religious, ideological, social and behavioral norms are often compelling in terms of their capacity to achieve primary health outcomes, they may also create unquantified downstream benefits, or constitute potential liabilities, from the diplomatic and foreign policy perspectives. Although it is critically important for such initiatives to attempt to optimize outcomes such as quality-adjusted life years (QALYs), their design and delivery needs to be carefully evaluated in order to ensure that (1) these goals are not being achieved at the cost of foreign policy, diplomatic, international relations, or broader global strategic objectives, and (2) their potential to achieve such collateral objectives is optimized [16].

### Ranking global health programs from the foreign policy and diplomatic perspectives

The rise of a utilitarian approach to global health evaluation, as represented by tools such as cost-effectiveness analysis, has resulted in the predominant use of narrow, single-metric measures as exclusive barometers of global health program worth or value [17]. No international initiative, however, operates in isolation or without epistemic consequences [18]. In a variation on the McNamara Fallacy [19], such programs produce a range of downstream, collateral or indirect outcomes that are not adequately quantified -- and, therefore, considered to be non-existent. In the 21^st^ century, both foreign policymakers and global health professionals require an innovative *métier* that reflects these broader considerations, both (1) in order to portray global health expenditures as investments rather than costs, and (2) to “speak a language that people with power really understand” [20]; foreign assistance priorities and associated resource allocation decisions should, where possible, include consideration of the universal and fundamental aspirations of global political, security, and strategic affairs [21]. The recent emergence of the smart power paradigm [22] has, at least in part, been a product of this increased integration between foreign policy and global health initiatives [23], elevating global health to the status of a powerful diplomatic and foreign policy tool, rather than merely a humanitarian effort. There has been, to date, a “lack of ability” to demonstrate the effectiveness of foreign assistance programs in this context [24]. This paper outlines the development and application of just such a “diplomatic assessment” approach for global health programmes, adopting a term from the art of fencing, and eponymously entitled the Kevany Riposte, due to it’s conceptual basis as an innovative and interdisciplinary approach that challenges “siloed” [13] or “stovepiped” [25] perspectives. These efforts are based on the author’s prior identification of “Top Ten” lists for diplomatic effectiveness [25], and as applied to a retrospective and collective assessment of PEPFAR and Global Fund-supported HIV/AIDS initiatives in South Africa, Sudan, Kenya, South Sudan, Zimbabwe, Tanzania, Iraq, Afghanistan, Egypt, and elsewhere, in order, for the first time, to assess intervention program threats and advantages from this broader perspective.

## Methods

### Defining “HIV/AIDS initiatives”

Community-based behavioral, educational, and diagnostic initiatives form an innovative and essential part of both the Global Fund and PEPFAR’s HIV/AIDS response paradigms [26,27]. Such programs provide, amongst other features, technical assistance to strengthen prevention programming; community support mechanisms; referral systems; and other behavioral and community-based health promotion and educational initiatives [28]. Within this broader operational context, HIV Counseling & Testing (HCT) based around “wellness days”, “community mobilization”, and “post-test support services”, amongst other forms of service delivery, are provided in community venues in order to increase, for example, numbers of persons tested for HIV/AIDS provided with personalized support and guidance regarding behavioral risk-reduction, as well as raising community awareness and engagement. In the health systems strengthening context, related interventions also contribute to capacity building (e.g. the provision of district-level trainers to implement “Prevention with Positives” programs) [29]. For the purposes of this paper, “HIV/AIDS interventions”, “HIV/AIDS programmes”, and “HIV/AIDS initiatives” therefore refer to such community-based, multi-level HIV/AIDS responses, including *combined* diagnostic, prevention, behavioural, and health system strengthening programs under the auspices of Project Accept [30], the Global Fund, and PEPFAR South Africa [31], whilst excluding therapeutic programs such as antiretroviral treatment and surgical interventions such as voluntary adult male circumcision.

### Kevany Riposte and K-Score development

Systems by which to quantify the latent and potential global health diplomacy and foreign policy value of global health programmes were developed based on previously-identified criteria for “global health diplomacy” and “global health and foreign policy” appropriateness and effectiveness in global health program design, delivery and evaluation [32]. Originally presented in the form of two “Top Ten” tables -- representative of those features or characteristics of global health programs identified as effective in the foreign policy or diplomatic contexts, respectively, in the related literature -- classifications were adapted to Excel-based questionnaire and scoring formats (see Additional file 1 Annex 1: Diplomatic Assessment Questionnaire and Scoring Tool). Development of the Kevany Riposte and K-Score also drew on the author’s prior contributions to the development and utilization of related designs, in particular the Global Fund’s Routine Service Quality Assessment (RSQA) and On-Site Data Verification (OSDV) tools [33]. The results presented in this paper relate specifically to “diplomatic”, rather than “foreign policy”, considerations.

### Implementation & utilization

Diplomatic assessments of HIV/AIDS initiatives were based on the retrospective and collective assessment of the author’s field deployments for diplomatic monitoring & evaluation, cost-effectiveness, and quality assurance purposes on behalf of the Global Fund, the United Nations Development Program, PEPFAR implementing organizations (e.g. the International Training and Education Center for Health), the University of California, San Francisco, and the Project Accept community-based voluntary counseling and testing intervention (as implemented under the United States’ National Institutes for Mental Health) between 2007 and 2014. Related on-site engagements included field missions to service delivery points (e.g. mobile HIV/AIDS counseling and testing centers in the Northwest Province of South Africa for PEPFAR and in Sudan and South Sudan for the Global Fund) and engagements and liaisons with key governmental and non-governmental organizations and individuals (e.g. the South African, Sudanese and South Sudanese Ministries of Health on behalf of the Global Fund). Field-level and on-site observations conducted to inform assessment responses were undertaken, where possible, with the assistance of an interview guide (see Additional file 2 Annex 2: Interview and Assessment Guide for Diplomatic and Foreign Policy Assessments) and were complemented, where necessary, by follow-up questions via e-mail exchanges and teleconferences with key managerial and field staff. Further on-site and desk research (e.g. review of intervention protocols and standard operating procedures) was also conducted to complete remaining questionnaire responses, as necessary.

### Defining assessment classifications & sub-classifications

The primary classifications employed in the Kevany Riposte and K-Score were based directly on previously-identified “Top Ten” criteria keywords (e.g. “communications”; “adaptability”), as described above. Related sub-classifications were based on interpretations and descriptions of each primary classification in the literature (e.g. the “adaptability” classification was associated with themes of “responsiveness to health needs”, “responsiveness to non-health needs”, “recipient-led program design”, and “recipient-led resource allocation”). For purposes of both detail and consistency, four such sub-classifications were developed for each classification, resulting in 40 assessment questions. In turn, these sub-classifications were expanded and articulated in the form of specific thematic questions to be addressed to relevant project staff, including program managers, field officers, and other project personnel, designed to be relevant to project activities at the individual, intervention and policy levels (see below).

### Policy, intervention and individual level assessment dimensions

As a result of the broad range of issues related to the diplomatically-effective delivery of global health programs, the above assessment procedures included implicit consideration of three main evaluation dimensions: (1) policy level (e.g. whether donor organization guidelines addressed relevant practices & procedures), (2) intervention level (e.g. whether intervention protocol and standard operating procedures were appropriately designed from the diplomatic perspective), and (3) individual level performance and responsibilities (e.g. sensitization of national and international project staff to consideration of broader local, national, and international strategic environments). Sub-classification questions were designed to be informed by, and completed based on consideration of, these three constructs.

### Responses, scoring and comments

Response options to sub-classification questions were divided into six categories: “highly advantageous”, “moderately advantageous”, “neutral, not relevant, or not considered”, “not applicable”, “potential moderate threat”, and “potential significant threat”, in accordance with level of alignment with associated diplomatic goals and principles (Table 1). These response categories were associated with scores of +2, +1, zero (for both “neutral, not relevant, or not considered” and “not applicable”), -1, and -2, respectively. Sub-classification scores were then aggregated and averaged in order to provide an overall classification score. No weighting was attached to different classifications or sub-classifications, based on the assumption that all classifications were of equal value or importance from the diplomatic perspective. Scope for additional comments, narrative descriptions, and categorization justification was also included at the assessment and evaluation stage in order to provide further explanation, as necessary, for scoring decisions. Finally, all classification scores were aggregated and averaged in order to determine an overall intervention-specific diplomatic assessment rating, representative of the collective programs under review, in the diplomatic context (the “K-Score”).

**Table 1:** Scoring and Results Classifications for “Top Ten” Criteria

Classification	Interpretation	Score
Highly advantageous	*Intervention program displays clear and significant value from the diplomatic or foreign policy perspective.*	**+2**
Moderately advantageous	*Intervention program displays some strengths in advancing diplomatic or foreign policy goals.*	**+1**
Acceptable, neutral, or not relevant	*Intervention attains diplomatic or foreign policy minimum standards.*	**0**
Not applicable	*Intervention program does not operate in the context of this classification (or sub-classification).*	**0**
Potential moderate threat	*Intervention program may constitute a threat to diplomatic or foreign policy goals.*	**−1**
Potential significant treat	*Intervention program constitutes a clear and significant threat from the diplomatic or foreign policy perspective.*	**−2**

## Program performance from the diplomatic perspective

### Overall diplomatic assessment results

**Fig. 1:**
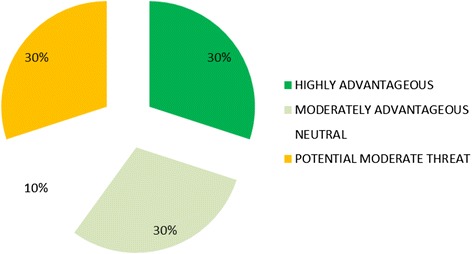
Diplomatic Advantages & Threats of Selected HIV/AIDS Initiatives

Diplomatic assessment ratings at the classification and sub-classification levels are presented in Table 2 and Additional file 1 Annex 1. The HIV/AIDS initiatives under review were collectively found to be “moderately advantageous” from the diplomatic perspective, attaining an overall average score of +1 across diplomatic assessment classifications. This included three “highly advantageous”, three “moderately advantageous”, one “neutral”, and three “potential moderate threat” classification scores (Fig. 1).

**Table 2:** Diplomatic Assessment Results

Classification	Sub-classification 1	Sub-classification 2	Sub-classification 3	Sub-classification 4	Score	Rating
Neutrality	*Cultural*	*Social*	*Religious*	*Other*		
Neutrality Score	−1	−1	−2	2	−1	POTENTIAL MODERATE THREAT
Visibility	*Appropriate Branding*	*Safety & Security*	*National Linkages*	*Visibility through Communications*		
Visibility Score	2	1	1	2	2	HIGHLY ADVANTAGEOUS
Sustainability	*Sustainability*	*Transferability*	*Intervention Type*	*Forward-Looking Commitments*		
Sustainability Score	−2	1	2	2	1	MODERATELY ADVANTAGEOUS
Effectiveness	*Effectiveness*	*Constrained Budgets*	*Cost-Effectiveness*	*Academic Evidence*		
Effectiveness Score	2	−1	−2	2	0	NEUTRAL
Adaptability	*Responsiveness to Health Needs*	*Responsiveness to Non-Health Needs*	*Recipient-Led Program Design*	*Recipient-Led Resource Allocation*		
Adaptability Score	1	2	2	2	2	HIGHLY ADVANTAGEOUS
Accountability	*Contributions to M&E Systems*	*Production of Verifiable Results*	*Presentation of Health & Non-Health Achievements*	*Combating Corruption and Increasing Transparency*		
Accountability Score	2	2	1	−1	1	MODERATELY ADVANTAGEOUS
Partnerships	*Reference to Standards of International Interaction*	*Building International Alliances*	*Interaction Coordinating Initiatives*	*Sub-National Partnerships*		
Partnerships Score	0	2	2	2	2	HIGHLY ADVANTAGEOUS
Economic, Political, Environmental and Social (EPES) Effects	*Economic Growth*	*Political Stability*	*Social Evolution*	*Environmental Impact*		
EPES Effects Score	2	1	2	−2	1	MODERATELY ADVANTAGEOUS
Interdependence	*Organizational Relationships*	*Staff Safety*	*Mission Statements*	*Joint Agenda Accomplishment*		
Interdependence Score	−1	0	−1	−1	−1	POTENTIAL MODERATE THREAT
Training	*Staff Selection*	*Staff Recognition*	*Education on Strategic Themes*	*Diplomatic Risks and Benefits*		
Training Score	−2	1	−1	−1	−1	POTENTIAL MODERATE THREAT
Overall K-Score & Assessment					1	MODERATELY ADVANTAGEOUS

### “Highly advantageous” classifications

HIV/AIDS initiatives were considered to be “highly advantageous” under the diplomatic assessment classifications of “visibility”, “adaptability”, and “partnerships”. These results are in keeping with prior findings related to both the structure and “unintended consequences” of HIV/AIDS intervention programs [18]. For example, the extensive intervention adaptability of related HIV/AIDS interventions has been documented elsewhere [34], including the importance of intervention visibility and communications through revisions to key practices (e.g. evolving intervention “branding” and terminology to fit with local cultures and social norms) and community mobilization activities. Similarly, the generation of international partnerships through collaborations based on international HIV/AIDS initiatives is in keeping with prior findings related to the documented success of academic and inter-governmental collaborations under both the Global Fund and PEPFAR [35].

### “Moderately advantageous” classifications

HIV/AIDS initiatives under review were found to be “moderately advantageous”, from the diplomatic perspective, in terms of “sustainability”, “accountability”, and “economic, political, environmental and social effects”. These findings are also supported by reference to related studies. For example, positive assessments for sustainability were driven by intervention strengths in the context of “country ownership” [36] and transferability to local actors, while diplomatic advantages in the “accountability” context are aligned with previously-documented strengths of PEPFAR and the Global Fund in monitoring and evaluation and other quality assurance and control systems [37]. Similarly, positive economic, political, and social outcomes were driven by the widespread provision of social and economic support services (such as “Back to Work” or income-generating horticultural schemes), which were frequently attached to those primary service delivery components under review [30].

### “Neutral” classifications

HIV/AIDS interventions were classified as “neutral” from the diplomatic perspective in the context of “effectiveness”. The limited consideration of budgetary constraints after the intervention or programmatic support period, combined with the high demand for affordable and cost-effective health care delivery and financing strategies in recipient countries, are current causes of concern throughout both the global health and international development contexts [38], most particularly in the current global recession era. In this context, the increased use of both health and non-health effectiveness and cost-effectiveness information as a key component of intervention design and delivery on a *prima facie* rather than a *post-hoc* basis may significantly strengthen intervention design and delivery [39] in this regard.

### “Potential moderate threat” classifications

Potential moderate threats to diplomatic considerations, interests and outcomes at both recipient and donor levels related to “neutrality”, “interdependence”, and “training”. These findings are, once again, in keeping with a range of contemporary recommendations on the design and delivery of global health intervention programs. For example, in the context of neutrality, the imposition of international cultural, social and religious standards and norms on recipient societies, often without appropriate levels of consultation at the local and community levels, may represent a threat both to successful program implementation *and* to international relations [34]. Conversely, a lack of awareness of the strategic implications of global health’s resource allocation decisions may mean that foreign assistance can unintentionally support extremist organizations [40]. Similarly, in the context of “interdependence”, a lack of organizational and operational coordination and alignment between health and non-health initiatives related to broader strategic and international affairs considerations has been identified as a possible source of conflict between health and diplomatic goals [41]. Finally, in the context of “training”, limited levels of broader political education and awareness in global health practitioners operating in international environments has been identified as a key gap at the individual capacity level [42,43].

### Potential significant threats at the sub-classification level

Although no classifications were rated as a “potential significant threat” from the diplomatic perspective, specific sub-classifications recording this result are highlighted here. These include programmatic threats to religious neutrality (under the “neutrality” classification), limited assurances and planning regarding funding sustainability (under the “sustainability” classification), limited or no *prima facie* use of health and non-health cost-effectiveness findings (under the “effectiveness” classification, and as described above), limited or no consideration of environmental impact (under the “economic, political, environmental and social effects” classification), and inadequate provision of broader diplomatic training, awareness, and formalization of related roles and responsibilities (under the “training” classification). Strategies to address these potentially significant diplomatic threats have been presented, in recent years, via innovative recommendations on the 21^st^ century design and delivery of global health service delivery [13].

## Interpretation & conclusions

### Key findings

For the first time, a global health intervention has been assessed through the lens, and from the perspective, of diplomatic appropriateness, sensitivity and effectiveness. HIV/AIDS initiatives were found to score positively in terms of diplomatic effectiveness, whilst also evincing both (1) potential areas of improvement and (2) a limited number of potential diplomatic threats. These findings may represent significant considerations for policymakers both within and beyond global health, who are now equipped to determine the value, worth or risk of global health investments beyond the narrow metrics employed by traditional monitoring and evaluation or cost-effectiveness analyses associated with a narrow (and often exclusively medical) selection of outcomes, outputs and impact assessments [44]. These results should, nonetheless, still be considered in conjunction with traditional measures of program effectiveness or efficiency in order to determine associated resource allocation and implementation decisions: low levels of intervention cost-effectiveness, for example, may be offset by significant intervention returns or value at the diplomatic level [35].

### Enlightened strategic & resource allocation decisions: beyond cost-effectiveness

The results presented here are designed for use and reference at both the global health policy level and across the broader *milieu* of bilateral and multilateral foreign policymaking and practice. For example, within global health and development, these results may help to inform “enlightened” resource allocation decisions beyond the “potentially flawed” [35] reliance on cost-effectiveness analyses as the exclusive determinant of global health program worth or value. Perhaps even more importantly, the interpretation and use of these results at the diplomatic and foreign policy level presents a range of opportunities for policymakers to leverage, design, and refine global health and other development initiatives for the purposes of foreign policy and diplomacy [45].

### “Smart power” and “smart global health”

At its most optimal, the Kevany Riposte and the K-Score may help both health and non-health, bilateral and multilateral organizations to manipulate aid in order to substitute for, offset, complement, or support the use of hard power in favour of smart power options, via the creation of a new “stage” in the escalation of international engagements, in keeping with 21^st^ Century standards of acceptability for, and effectiveness of, international military interventions [46,47]. A new step in the “escalation hierarchy” amongst traditional and accepted stratifications such as neutrality, diplomacy, soft power and hard power [48] would be epitomized by such smart global health initiatives [45]. This may, in turn, bring about (1) a transfer or collaboration of resources from hard to smart international initiatives operating under “military umbrellas” [46] and, where feasible and appropriate, (2) the increased or enhanced use by military forces and related organizations of smart global health systems to pursue foreign policy and strategic prerogatives [49].

### Utilization in the international intelligence context

The employment of global health programs for strategic political ends in an unstructured manner has, in the past, put global health workers at a security risk by association, regardless of whether or not individual- or organizational-level activities are in fact related to such ostensibly extraneous objectives [50]. At the same time, Western powers are increasingly open to the use of innovative, collaborative and interdisciplinary efforts to resolve contemporary international affairs and security challenges [51], against which conventional response systems have faced significant challenges. In this highly nuanced, complex, and occasionally clandestine context, non-health dividends may be attained if global health programs are selected, designed and delivered in a manner that bears in mind potential international conflict resolution, cooperation, and security goals *as well as* primary health and development outcomes [52]; past research has suggested that locating highly-diplomatic global health projects in extremist regions, as informed by the “geo-strategic considerations” classification, provides meaningful alternatives to political or other forms of extremism [53]. In this context, the Kevany Riposte and the K-Score may offer standards by which international agencies such as the United Kingdom’s Security Service,(MI5), the European Union’s Intelligence Analysis Centre [54], and the United States’ Central Intelligence Agency (CIA) liaise with both donor, supranational, and recipient departments of international development and health in a fashion that both (1) reduces threats to aid workers and (2) integrates multifarious dimensions to global health programs which are, in turn, (3) conducted in a style acceptable and transparent to recipient country governments. Such innovative collaborations, instead of acquiescing to demands that global health funding be transferred to defense [55], stand to achieve both altruistic, intelligence, and security goals simultaneously. Perhaps most importantly, employment of assessment systems under the Kevany Riposte in this context will also help to ensure that global health programs do not inadvertently *harm* international security by providing aid, health, or other financial support to extremist organizations [56]. Notwithstanding these other potential gains, traditional (and possibly flawed) approaches to intelligence gathering through international development initiatives [57] stand to be both improved and made more effective by the application of relevant criteria, models, procedures and standards to both organizational and individual-level activities and liaisons in this context.

### Limitations

The absence of comparator results from other global health intervention programs is a key limitation of this work. The generation of relevant comparable “K-Scores” in different settings (e.g. the diplomatic effectiveness of tuberculosis treatment programs in Iraq or malaria prevention initiatives in Afghanistan, as described elsewhere by the author) [17,52] may, as with the results of cost-effectiveness analyses, generate opportunities for intervention ranking or league tables [58] from the diplomatic or foreign policy perspective. For example, comparisons with HIV/AIDS interventions excluded from this review (e.g. antiretroviral treatment or male circumcision) might provide useful information to policymakers regarding related resources allocation decisions. This review should, therefore, be characterized and interpreted as a pilot initiative, based on which, in future, Kevany Riposte systems, scope, and results may be further refined and applied by larger teams. In addition, future efforts might also (1) consider dividing the system’s structure and results across the three major evaluation dimensions outlined above (i.e. individual, policy and intervention levels) in a more explicit fashion, (2) correlate health and diplomatic outcomes (see below), and (3) attempt to further describe the political and operational mechanisms by which the results of diplomatic and foreign policy evaluations may be translated from findings such as those presented here into policy, and thence into practice [59–61] (see below).

### Plotting health effectiveness against intervention effectiveness

A related area of potential interest and inquiry to organizations such as the United States’ Centers for Disease Control and Prevention (CDC) is consideration of the effects of diplomatically “highly advantageous” global health interventions on health outcomes. Do “more diplomatic” global health interventions relate to improved health outcomes – or vice versa – or both? Such findings, though beyond the scope of this paper, might be determined through cross-referencing intervention medical efficacy and effectiveness against diplomatic assessment values. Though direction of causality may be challenging to prove, the establishment of such relationships may help to further explain the manifold connections between diplomatically and medically successful global health interventions [62].

### Recommendations

Global health interventions and related Global Fund and PEPFAR-supported programs have the potential to be of significant importance in alleviating developing countries from the worst effects of communicable and non-communicable disease and ill-health. More broadly, such interventions may also have the potential to advance diplomatic considerations related to the interests of both donor and recipient countries, as well as national and international, health and non-health, goals and initiatives, such as strategic and security concerns. In order to optimize the potential future impact of these latter dimensions, and based on the results presented here, related recommendations include (1) consideration of the redesign of HIV/AIDS initiatives in the context of training, organizational interdependence, and neutrality, whilst also addressing the specific “potential significant threats” at the sub-classification level described above; (2) the further development of intervention sustainability, accountability, and latent political, economic, social and environmental potential; (3) the development and *prima facie* integration of intervention health and non-health effectiveness findings into intervention program design and delivery; (4) building on the successes and diplomatic advantages associated with intervention visibility, adaptability, and partnership development; and (5) leveraging these latent diplomatic assets, at the individual, intervention and policy levels, in order to address broader national and international strategic concerns.

### Next steps: utilization of results and “evidence into policy & practice”

The identification of five main opportunities and mechanisms for utilization of the results of the Kevany Riposte and K-Score have previously been identified by the author as (1) training, (2) evaluation, (3) resource allocation, (4) funding, and (5) military and international security considerations [25]. A range of *realpolitik* scenarios for such applications are conceivable. In one possible example, widespread diplomatic reviews conducted under the auspices of organizations such as the Office of Global Health Diplomacy, the United Kingdom’s Royal Institute for International Affairs, or the European Union’s External Action Service, might provide a detailed picture of the comparative worth of global health interventions, from the diplomatic and foreign policy perspectives, across a range of key settings, population groups, and regions around the world. These results might then be overlaid, with the assistance of donor and recipient country foreign policymakers or professional diplomats, with global diplomatic, political, foreign policy or strategic needs and threats in order to determine both (1) overall and targeted global health investments, (2) geo-strategic and demographic focus, and (3) intervention program selection, whilst (4) better aligning interventions with broader strategic considerations and the work of non-health international initiatives and organizations. In this way, the smart use of global health initiatives may provide a meaningful and effective alternative or complement to other forms of international intervention on the world stage, advancing both health and non-health goals [63].

## Additional files

Additional file 1:
**Annex 1 Diplomatic Assessment Questionnaire and Scoring Tool.**
Additional file 2:
**Annex 2 Interview and assessment guide for diplomatic and foreign policy assessments.**
