# Supplementary material for: Diplomatic advantages and threats in global health program selection, design, delivery and implementation: development and application of the Kevany Riposte
Source: Global Health. 2015 May 27;11:22. doi: 10.1186/s12992-015-0108-x (PMC4470080; doi:10.1186/s12992-015-0108-x)
Supplement: Additional file 2: — Annex 2 Interview and assessment guide for diplomatic and foreign policy assessments. [file 12992_2015_108_MOESM2_ESM.docx]

**Interview and Assessment Guide for Diplomatic and Foreign Policy Assessments**

***Key Staff:*** Staff selected to respond to the diplomatic and foreign policy assessment questions should be chosen on the basis of (1) availability; (2) knowledge and understanding of program or project design and delivery; (3) past associations with the project or intervention; and (4) willingness to discus and comment on broader aspects of the project beyond health issues and outcomes.

***Choice of Respondents:*** The choice of respondents for each section of the assessment tool may also be informed by the “assessment level” categories. For example, those questions relevant to the “individual” level may best be resented to filed or project staff, while those questions concerned with “Intervention” or “Policy” level may best be presented to senior in-country or headquarters management (or, if relevant, academic) personnel

***Document Review and Site Visits:*** It is anticipated that not all staff will have sufficient knowledge or understanding of the foreign policy or diplomatic contexts in which this intervention operates to answer all questions. In such cases, interview responses should be complemented with desk reviews of relevant project planning and delivery documents (e.g. standard operating procedures and project protocols). Where possible, these desk reviews should be further verified through site visits to project service delivery points.

***Interview Structure and Duration:*** Interviews are to be conducted on an ad-hoc, informal basis based on the availability of key program staff. Interviews are anticipated to take no more than one to two hours of respondent time, and can be conducted in the field or office environments, as appropriate. Where possible, interview responses will be entered directly into the assessment tool spreadsheet with associated comments and response ratings. These will subsequently be translated into scores for each criterion and at the policy, individual and intervention levels.

***Spreadsheet Completion, Including Cover and Summary Pages:*** Spreadsheet completion and review will take place after completion of the field and office-level questionnaires. Key information for these sections will be based on academic and program document review (e.g. program effectiveness and funding history). These results will then form the basis of the results to be presented in Tables 2 and 3 (see draft manuscript outline).
